# Advancing precision immuno-oncology in melanoma: the synergistic convergence of personalized neoantigen vaccines and multi-omics biomarker profiling

**DOI:** 10.3389/fimmu.2026.1831047

**Published:** 2026-04-29

**Authors:** Yulong Hou, Can Chen, Xi Chen

**Affiliations:** 1 Huzhou Central Hospital, Fifth School of Clinical Medicine of Zhejiang Chinese Medical University, Huzhou, China; 2 Huzhou Central Hospital, Affiliated Central Hospital of Huzhou University, Huzhou, China; 3 Department of Dermatology, First Affiliated Hospital of Huzhou University, Huzhou, China

**Keywords:** biomarkers, immune checkpoint inhibitors (ICIs), melanoma, multi-omics, personalized neoantigen vaccine, therapeutic resistance

## Abstract

Cutaneous melanoma represents a paradigm of immunological complexity, where multifactorial primary and acquired resistance often undermine the clinical efficacy of conventional immune checkpoint blockades and targeted therapies. This review provides a comprehensive analysis of the strategic shift toward precision immuno-oncology, focusing on the mechanistic synergy between personalized neoantigen-directed vaccines and integrated multi-omics profiling. Rather than viewing these as independent pillars, we propose an integrated framework where multi-omics data functions as the indispensable ‘operating system’ that drives the entire lifecycle of neoantigen vaccines. By targeting patient-specific somatic mutations, bespoke vaccines are capable of orchestrating *de novo*, high-avidity T-cell responses with superior specificity and minimal off-target toxicity. We evaluate the clinical evolution and immunological foundations of advanced vaccine platforms—including mRNA, synthetic long peptides (SLPs), and engineered exosome-based systems—highlighting their transformative potential in the neoadjuvant and adjuvant settings. Central to this paradigm is the deployment of a robust multi-omics ecosystem (encompassing genomics, transcriptomics, epigenomics, proteomics, and the host microbiome) to decipher the dynamic landscape of the melanoma tumor microenvironment (TME). Facilitated by artificial intelligence (AI) and real-time liquid biopsy monitoring, this framework enables an iterative, biologically informed feedback loop for adaptive clinical management. We emphasize that the integration of personalized vaccines with immune checkpoint inhibitors (ICIs) and MAPK pathway inhibitors is essential to dismantle resistance barriers, providing a definitive roadmap for achieving durable clinical remission and curative outcomes in the era of personalized oncology.

## 1 Introduction

Cutaneous melanoma remains a highly aggressive malignancy with a rising global incidence (1). Historically, the prognosis for advanced stages was dismal, with five-year survival rates as low as 25% due to the disease’s inherent resistance to conventional chemotherapy and radiotherapy (2, 3).

The last decade has marked a revolutionary shift from non-specific cytotoxic approaches to a new era of precision medicine (4, 5). This revolution is driven by two synergistic pillars: molecularly targeted therapy and cancer immunotherapy (6). Agents targeting the BRAF/MEK pathways and immune checkpoint inhibitors (ICIs) targeting the CTLA-4 and PD-1/PD-L1 axes have provided unprecedented durable responses (7–10). While these modalities have become the standard of care (4, 11), primary and acquired resistance, along with the lack of reliable predictive biomarkers, remain significant clinical hurdles (8, 10, 11).

Current therapeutic paradigms often fall short of fully personalizing treatment to the unique molecular and immunological architecture of an individual’s tumor (12). This gap has catalyzed the shift towards true precision immuno-oncology, centering on the convergence of personalized neoantigen-directed therapy and multi-omics biomarker profiling (13, 14). Personalized vaccines address tumor heterogeneity by targeting patient-specific mutations, offering potent anti-tumor immunity with minimal off-target effects (13).

The successful deployment of these bespoke therapies necessitates sophisticated biomarker-driven frameworks to decipher the predictive and prognostic code (12, 14). This review explores the transformative journey from empirical combinations toward biomarker-guided synergy, providing a roadmap for next-generation personalized management defined by a precise molecular and immunological blueprint (13, 15).

## 2 Neoantigen-directed vaccines: immunological foundations and clinical evolution

The transition towards precision immuno-oncology is fundamentally embodied by the development of personalized neoantigen-directed vaccines. This paradigm shift is essentially a biological and computational fusion, where the ‘bespoke’ nature of the vaccine is predicated on high-dimensional omics inputs. This approach shifts the paradigm from non-specific immune activation to the targeted induction of T-cell responses against truly tumor-specific mutations, offering a tailored therapeutic strategy with the potential for high efficacy and minimal off-target toxicity (13, 16). The conceptual foundation rests on the identification of immunogenic neoantigens—unique peptides derived from somatic mutations that are presented on the tumor cell surface by major histocompatibility complex (MHC) molecules. These neoantigens are absent from normal tissues, making them ideal targets for immune recognition and attack. The high tumor mutational burden (TMB) characteristic of melanoma, often resulting from ultraviolet light-induced DNA damage, provides a rich repository of candidate mutations, positioning this malignancy as a prime candidate for such personalized immunotherapy (17).

**Figure 1:**
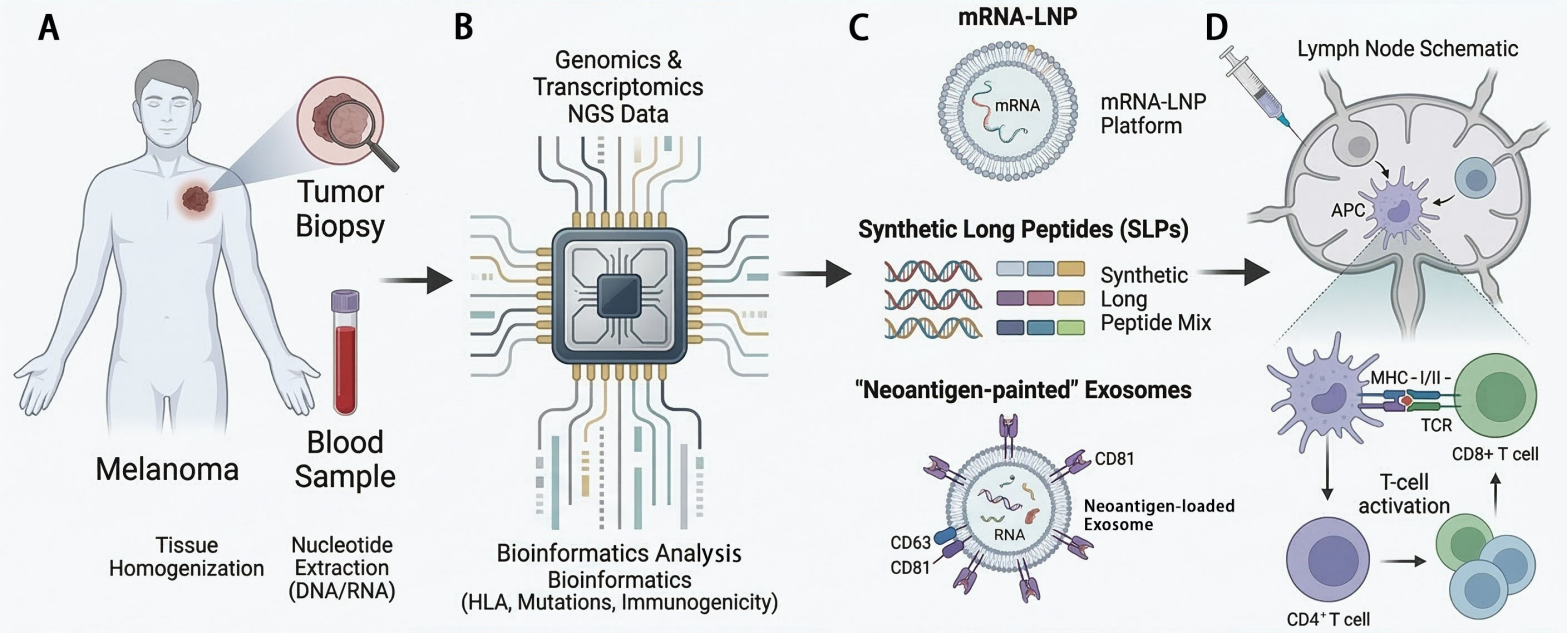
Personalized neoantigen vaccine: a comprehensive development and implementation pipeline **(A)** Collection of tumor biopsies and peripheral blood to identify patient-specific somatic mutations through whole-exome and RNA sequencing. **(B)** Deployment of advanced bioinformatics pipelines and AI algorithms to perform HLA typing, mutation calling, and neoepitope binding prediction to prioritize highly immunogenic candidates. **(C)** Integration of selected neoantigens into diverse delivery systems, including mRNA encapsulated in lipid nanoparticles (LNPs), synthetic long peptides (SLPs), and engineered “neoantigen-painted” exosomes designed to enhance lymph node homing. **(D)** Clinical administration of the vaccine to prime *de novo* T-cell responses and promote the expansion of tumor-specific CD8+ and CD4+ T-cell clones.

Translating this concept into clinical reality requires a multi-step pipeline beginning with tumor and normal tissue sequencing to identify somatic mutations. Subsequent *in silico* prediction algorithms are employed to forecast which mutated peptides will be processed and presented by the patient’s specific HLA alleles, a critical step for prioritizing candidate neoantigens (18, 19). The validity of this approach was underscored by a multi-omics study which successfully identified naturally presented mutated HLA ligands in melanoma, in stark contrast to malignancies like hepatocellular carcinoma with lower TMB, highlighting the particular suitability of melanoma for exome-derived neoantigen vaccines (17). This pipeline represents an iterative multi-omics feedback loop, where genomic depth identifies potential targets and transcriptomic validation ensures their biological relevance within the tumor microenvironment (TME) (**Figure 1**). The selected neoantigens are then incorporated into a vaccine platform. Early cancer vaccine strategies often targeted shared tumor-associated antigens (TAAs), but these faced challenges related to immune tolerance and on-target, off-tumor toxicity. Neoantigen vaccines, by targeting truly tumor-exclusive epitopes, circumvent these limitations and can induce *de novo* high-avidity T-cell responses (13, 20).

The clinical landscape is now populated by diverse and innovative vaccine platforms, each with distinct advantages. Synthetic long peptides (SLPs) and mRNA vaccines are among the most advanced (18). Peptide-based vaccines, such as the personalized neoepitope vaccine EVX-01 evaluated in combination with pembrolizumab in the KEYNOTE-D36 trial for advanced melanoma, deliver defined antigen sequences directly (21). mRNA vaccines offer a flexible platform where the RNA sequence encoding the neoantigen is delivered, enabling endogenous antigen production and presentation that may more closely mimic natural infection, potentially broadening the immune response (16, 18). Beyond these, novel delivery and formulation strategies are rapidly emerging to enhance immunogenicity. These include neoantigen-displaying self-assembling protein nanoparticles, which in preclinical models have demonstrated the ability to elicit potent CD4+ T-cell responses and inhibit melanoma growth (22), and exosome-based platforms where neoantigens are anchored to serum exosomes to promote lymph node homing and dendritic cells (DCs) uptake, significantly amplifying T-cell responses *in vivo* (23). Nanotechnology plays a pivotal role, with systems like cationic lipo-polymer complexes serving as flexible platforms for co-delivering antigens and immunomodulators (e.g., anti-CD28 antibodies) to tailor specific immune responses (24), and microneedle arrays designed for transdermal delivery of nanomotor-based vaccines combined with chemotherapeutic agents, offering a localized and synergistic treatment modality (25).

The clinical evaluation of these vaccines is increasingly focused on rational combination strategies, particularly with ICIs. The biological rationale is compelling: a neoantigen vaccine aims to expand the pool of tumor-specific T cells, while ICIs remove inhibitory signals to unleash their effector function. This synergy has been demonstrated in numerous preclinical models. For instance, complete tumor eradication was achieved in murine colon cancer models using neoantigen-painted exosome vaccines combined with anti-PD-1 therapy (23). Similarly, in melanoma models, combinations of photodynamic therapy with a flagellin-adjuvanted peptide vaccine significantly potentiated the efficacy of PD-1 blockade (26). Whole tumor cell-based vaccines, engineered to be safer and more immunogenic—such as a frozen dying tumor cell (FDT) vaccine—have also shown that when combined with cytokines and ICIs, they can achieve 100% tumor eradication in preclinical settings (27).

Early-phase human trials are translating this preclinical synergy into encouraging clinical signals. The KEYNOTE-D36 trial is a prime example, testing whether the addition of the personalized peptide vaccine EVX-01 can convert initial stable or partial responses to pembrolizumab into deeper and more durable responses (21). Beyond fully personalized vaccines, off-the-shelf approaches targeting shared antigens continue to be explored. The telomerase-targeting long-peptide vaccine UV1, when combined with ipilimumab in melanoma patients, induced rapid and frequent T-cell responses that were shown to be remarkably durable and dynamic, persisting for up to 7.5 years in long-term follow-up (28). This study not only demonstrated the feasibility of inducing long-lived immune memory but also highlighted that the combination with checkpoint blockade can accelerate and enhance the vaccine-induced immune response kinetics (28) (**Table 1****).**

**Table 1:** Overview of leading neoantigen vaccine platforms and clinical trials in melanoma.

Vaccine platform	Representative agent/trial	Antigen targeting strategy	Key clinical/preclinical findings	Reference (year)
SLPs	EVX-01 (KEYNOTE-D36)	Personalized neoepitopes derived from somatic mutations	Evaluated in combination with pembrolizumab to convert initial stable or partial responses into deeper, durable responses	(21) [2022]
mRNA	Personalized mRNA vaccines	RNA sequence encoding neoantigens for endogenous production	Flexible platform mimicking natural infection; particularly recommended for “immune-cold” subtypes to ignite immune response	(16, 18) [2024, 2026]
Exosome-based	Neoantigen-painted exosomes	Surface-anchored neoantigen peptides on serum-derived exosomes	Promotes lymph node homing and enhances DC uptake; achieved complete tumor remission in murine models when combined with anti-PD-1	(23) [2023]
Nanoparti-cles	Self-assembling protein nanoparticles	Genetically engineered protein platforms (e.g., BP26) displaying tandem neoantigen repeats	Elicits potent neoantigen-specific CD4+ T-cell responses and significantly inhibits aggressive melanoma growth in preclinical models	(22) [2025]
Shared Antigen Peptides	UV1 (hTERT-targeting)	Long-peptide vaccine targeting the shared antigen telomerase	Induced remarkably durable and dynamic hTERT-specific T-cell responses persisting for up to 7.5 years in long-term follow-up	(28) [2022]

Despite this remarkable progress, the path from concept to widespread clinical reality is paved with significant challenges. The entire process—from tumor sequencing and neoantigen prediction to vaccine manufacturing and administration—is complex, time-consuming, and costly, presenting hurdles for scalability (19). Patient-specific factors, such as the genetic diversity of HLA alleles and the intrinsic heterogeneity of tumors, can limit the identification of immunogenic neoantigens for every individual (24). Furthermore, not all vaccine-induced T-cell responses translate into clinical tumor regression, underscoring the critical need for predictive biomarkers to identify patients most likely to benefit (18, 19). Bioinformatics is playing an increasingly vital role in addressing some of these challenges, not only in neoantigen prediction but also in stratifying patients. For instance, computational analysis of skin cutaneous melanoma has identified distinct immune subtypes, suggesting that patients with an immune-cold subtype (characterized by low immune activity) might derive particular benefit from an mRNA vaccine designed to ignite an immune response (29).

The current clinical reality of neoantigen-directed vaccines in melanoma is therefore one of promising transition. They have moved from a compelling theoretical concept to being evaluated in late-phase clinical trials, primarily in combination with established immunotherapies (18, 21). The focus has expanded beyond metastatic disease, with the neoadjuvant setting in stage III melanoma presenting a compelling opportunity for vaccine priming in the context of an intact tumor and lymphatic system (18). While standalone efficacy remains an area of active investigation, the predominant paradigm is one of combination, aiming to convert the modest activity of early-generation cancer vaccines into potent, synergistic regimens (16, 20). This evolving landscape sets the stage for the next critical step: integrating multi-omics biomarkers to refine neoantigen selection, predict responders, and guide the rational design of these increasingly sophisticated personalized immunotherapies.

## 3 Multi-omics biomarkers: deciphering the predictive and prognostic code

The journey towards rational combination strategies and optimized neoantigen vaccine design is critically dependent on the ability to accurately predict which patients will benefit and to understand the dynamic biological states that underlie response and resistance. This necessitates moving beyond single-dimensional analyses to a multi-omics framework that integrates genomic, transcriptomic, epigenomic, proteomic, metabolomic, and immunologic data (30–32). In this context, multi-omics biomarkers do not merely serve as passive indicators of disease state but act as the active blueprint for engineering and fine-tuning next-generation vaccines. In melanoma, where high heterogeneity and complex immune interactions define clinical outcomes, such a comprehensive approach is essential to decipher the predictive and prognostic code, transforming the current paradigm of combination immunotherapy from empirical to biomarker-informed (33, 34).

Genomic biomarkers represent the foundational layer, with BRAF V600 mutation status firmly established as a predictive biomarker for targeted therapy selection (33, 35). However, the predictive power of genomics for immunotherapy, including vaccines, extends beyond single driver mutations. TMB, while correlated with response to ICIs in some contexts, has not proven to be a standalone, robust predictor for melanoma, reflecting the complexity of antigen presentation and immune evasion (34, 36). In uveal melanoma, a distinct molecular landscape defined by mutations in *GNAQ*, *GNA11*, *BAP1*, *SF3B1*, and *EIF1AX* provides critical prognostic information and underscores the fundamental biologic differences that necessitate tailored biomarker and therapeutic approaches compared to cutaneous melanoma (37–39). The integration of germline genetic factors is also emerging as a component of a comprehensive biomarker profile, potentially influencing individual susceptibility and clinical course (40).

Transcending the static genomic code, transcriptomic and epigenomic analyses provide a dynamic readout of tumor and immune cell states. Gene expression profiling assays have been developed to improve risk stratification in primary melanoma, though their clinical integration for prognosis and sentinel lymph node prediction requires further validation (33, 41). More directly relevant to immunotherapy, transcriptomic signatures of an inflamed TME, characterized by expression of interferon-gamma related genes, cytotoxic T-cell markers, and antigen presentation machinery, have been associated with response to ICIs (34, 42). Recent studies integrating DNA methylome and transcriptome data from pre-treatment melanoma tissues have identified specific epigenetic and gene expression patterns correlating with anti-PD-1 response, highlighting the regulatory role of epigenetics in shaping the immunogenic phenotype of the tumor (43). Long non-coding RNAs (lncRNAs) and microRNAs (miRNAs) are additional layers of epigenetic and post-transcriptional regulation implicated in melanoma pathogenesis, progression, and therapy resistance, presenting a largely untapped reservoir for biomarker discovery (44–47).

The proteomic and metabolomic layers bridge genotype to phenotype and functional state. Serum biomarkers such as lactate dehydrogenase (LDH) and S100B have long been used for monitoring disease burden and prognosis, though with limited predictive value for therapy (48–50). Emerging proteomic analyses are identifying novel serum and tissue-based protein markers. For instance, soluble CD27 (sCD27) has been shown to differentially predict resistance to anti-PD-1 monotherapy but not to combination anti-PD-1/CTLA-4 therapy in metastatic melanoma, offering a potential tool for therapeutic escalation decisions (51). Sex-specific differences in serum cytokine levels (e.g., IL-4, IL-6, IL-10) have also been linked to ICI efficacy, underscoring the importance of host factors in biomarker profiles (52). At the cellular level, the metabolic fitness of DCs used in vaccine generation, specifically a shift towards glycolysis, has been correlated with poor patient overall survival, positioning immune-metabolic profiles as a crucial biomarker for cellular vaccine potency (53). Furthermore, enzymes like MTHFD1L and matrix metalloproteinases (e.g., MMP11, MMP14) are being investigated as prognostic markers and therapeutic targets linked to tumor progression and extracellular matrix remodeling (54, 55).

The paradigm of biomarker discovery has been revolutionized by liquid biopsy, which enables real-time, minimally invasive monitoring of tumor dynamics and treatment response. Circulating tumor DNA (ctDNA) is the most advanced liquid biopsy analyte, with compelling data supporting its utility. In the adjuvant setting for stage III melanoma, detection of ctDNA after surgery identifies patients at the highest risk of relapse and predicts worse overall survival, while longitudinal monitoring can provide early evidence of molecular response or recurrence during therapy (56, 57). In metastatic disease, a decrease in ctDNA variant allele fraction early during ICI treatment is strongly associated with improved progression-free and overall survival (56). Beyond ctDNA, circulating tumor cells (CTCs) and extracellular vesicles (EVs), including exosomes, carry proteins, nucleic acids, and lipids that reflect the tumor of origin and its interaction with the microenvironment, offering a rich source for multi-omic biomarker discovery (58–61). The analysis of EVs is particularly promising for capturing snapshots of intercellular communication and immune modulation (61). Serial liquid biopsies provide a real-time omics readout of vaccine-induced T-cell efficacy, allowing for the early identification of molecular escape and the subsequent adaptation of the vaccine’s antigenic cargo.

The immunome—the complete set of immune cell populations and their functional states—is arguably the most direct biomarker for immunotherapies. The density, location, and composition of tumor-infiltrating lymphocytes (TILs) have prognostic value and are under intense investigation as predictive biomarkers for ICIs and adoptive cell therapies (62, 63). High-dimensional analyses using mass cytometry and single-cell RNA sequencing are deconvoluting the immune microenvironment, revealing the significance of specific T-cell subsets, B cells, tertiary lymphoid structures, and innate immune cells like eosinophils and myeloid-derived suppressor cells (MDSCs) (30, 62, 64–66). Beyond mere T-cell infiltration, the spatial redistribution of immune cells into structured aggregates known as Tertiary Lymphoid Structures (TLS) has emerged as a cornerstone of the ‘hot’ tumor phenotype. These organized niches, characterized by B-cell-rich follicles and adjacent T-cell zones, serve as local command centers that facilitate efficient antigen presentation and the maturation of B cells into plasma cells (67) High-dimensional multi-omics studies have consistently demonstrated that the presence of TLS and robust B-cell/plasma cell signatures is strongly associated with superior progression-free survival and pathological response to ICI therapy in melanoma (67–69). These B-cell-rich microenvironments are thought to diversify the anti-tumor repertoire not only through direct antigen presentation but also via the production of tumor-reactive antibodies, thereby orchestrating a synergistic cellular and humoral immune response that is more potent than T-cell activity alone (69). The T-cell receptor (TCR) repertoire, analyzed through sequencing, provides a dynamic measure of antigen-specific T-cell clonal expansion, with early changes during ICI treatment showing predictive potential (70). Furthermore, the host microbiome, particularly the gut microbiota, has emerged as a powerful modulator of systemic immunity and ICI efficacy, with specific bacterial taxa and metabolites associated with clinical response, positioning it as a novel therapeutic co-driver and biomarker (71, 72).

**Figure 2:**
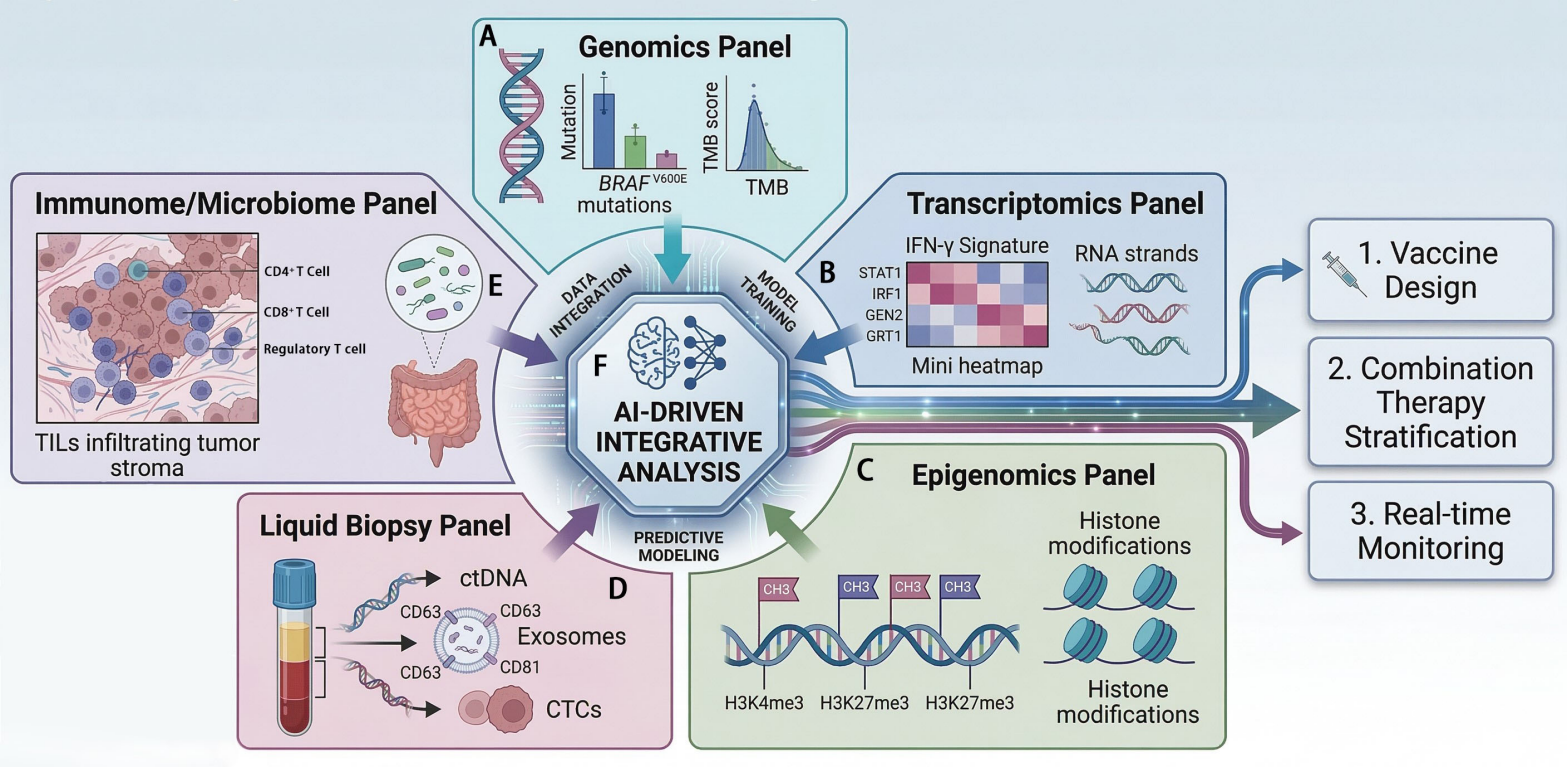
Integrated multi-omics evaluation ecosystem in melanoma. **(A)** Genomics Panel: Assessment of BRAF mutations and TMB as foundational indicators of treatment response. **(B)** Transcriptomics Panel: Evaluation of gene expression profiles and inflamed TME signatures, such as IFN-γ related genes. The mini heatmap symbolizes the integrated multi-omics signatures used for AI-driven patient stratification. **(C)** Epigenomics Panel: Identification of DNA methylation patterns and histone modifications that regulate the immunogenic phenotype of the tumor. **(D)** Liquid Biopsy Panel: Illustrates the isolation of ctDNA, exosomes, and CTCs. These components serve as vehicles for monitoring dynamic biomarkers, such as exosomal PD-L1 and CTC-surface PD-L1 expression, providing real-time insights into therapeutic response. **(E)** Immunome & Microbiome Panel: Analysis of TILs density and gut microbiota composition as systemic modulators of immunotherapy efficacy. **(F)** AI Core: Convergence of high-dimensional data into an integrated hub for predictive modeling, facilitating personalized vaccine design and therapeutic stratification.

Integrating these disparate omics layers is the central challenge and opportunity. Advanced computational approaches, including artificial intelligence (AI) and machine learning, are essential for identifying complex, multi-parametric signatures that outperform single biomarkers (73). Radiomics, which extracts high-dimensional data from medical images like CT and PET, can be combined with genomic and clinical data via AI to create non-invasive predictive models for treatment response and prognosis (73, 74). Similarly, integrative bioinformatics analyses are identifying novel gene signatures, such as those related to ferroptosis, that correlate with prognosis and may reveal new therapeutic vulnerabilities (75, 76). The goal is to move from a collection of individual biomarkers to an integrated biomarker ecosystem that can guide personalized treatment algorithms. This includes selecting patients for neoantigen vaccines based on their mutational landscape and HLA type, predicting vaccine efficacy through pre-vaccination immune and metabolic profiles, choosing optimal combination partners (e.g., anti-PD-1, anti-CTLA-4, targeted therapy) based on tumor-intrinsic and microenvironmental features, and monitoring therapeutic efficacy and emerging resistance through longitudinal liquid biopsies (34, 51, 53, 56) (**Figure 2****) (****Table 2**).

**Table 2:** Integrated multi-omics biomarkers for precision management in melanoma.

Omics layer	Specific biomarker(s)	Clinical utility	Specimen source	Reference (year)
Genomics	BRAF V600, TMB, GNAQ/GNA11	BRAF guides targeted therapy; GNAQ/GNA11 provides prognosis for uveal melanoma; TMB reflects immune evasion complexity	Tumor Tissue	(33–39) [2016–2026]
Transcriptomics	IFN-γ signature, STAT1 protein levels	IFN-γ related genes and STAT1high status predict superior response to ICIs compared to PD-L1 alone	Tumor Tissue	(34, 42, 43) [2017–2025]
Epigenomics	DNA methylation (e.g., SLC22A17), lncRNAs/miRNAs	Intragenic hypermethylation of SLC22A17 correlates with improved clinical outcomes and TME modulation	Tumor Tissue	(43–47) [2017–2025]
Proteomics	Soluble CD27 (sCD27), LDH, S100B, Cytokines (IL-6, IL-10)	sCD27 predicts resistance to anti-PD-1 monotherapy; LDH and S100B monitor disease burden and prognosis	Serum/Plasma	(48–52) [2010–2025]
Liquid Biopsy	ctDNA variant allele fraction, sEV-PD-L1	ctDNA clearance at 6 months predicts long-term clinical benefit; sEV-PD-L1 helps differentiate pseudoprogression	Plasma/Serum	(56, 57, 61) [2023–2024]
Microbiome	Gut microbiota composition (e.g., Anethole-associated taxa)	Specific bacterial taxa and metabolites associated with clinical response to both immunotherapy and targeted therapy	Fecal Samples	(71, 72) [2020–2025]

Despite the rapid advancement, significant hurdles remain for the clinical translation of multi-omics biomarkers. These include the need for standardized assays and cut-off values, validation in large, prospective, and heterogeneous cohorts, demonstration of clinical utility that improves patient outcomes, and addressing cost and accessibility issues (31, 33, 73). Furthermore, the field must contend with spatial and temporal heterogeneity, where a single tumor biopsy or a snapshot in time may not represent the entire disease biology (34, 77). The future of biomarker development lies in dynamic, multi-modal monitoring that combines tissue-based prognostic signatures with serial liquid biopsies and functional imaging, all interpreted through intelligent computational platforms. This holistic approach will be indispensable for realizing the full potential of personalized neoantigen vaccines and combination immuno-oncology, enabling the transition from a one-size-fits-all treatment model to a truly precision-guided, adaptive therapeutic strategy for melanoma.

## 4 Integrating multi-omics data for personalized vaccine design and treatment stratification

The transition from isolated biomarker discovery to their integrated application marks the critical evolution towards functional precision immuno-oncology. Overcoming the limitations of single-omics approaches requires a synergistic framework where genomic, transcriptomic, epigenomic, proteomic, and metabolomic layers are converged to construct a high-fidelity, dynamic portrait of an individual’s tumor and immune ecosystem. This integrative multi-omics paradigm is indispensable for two interconnected goals: the rational design of truly personalized neoantigen vaccines and the intelligent stratification of patients for optimal therapeutic combinations. It moves beyond static snapshots to model the complex biology determining vaccine immunogenicity and treatment responsiveness (78).

The foundation of neoantigen vaccine design rests on accurately identifying tumor-specific mutations and predicting which mutated peptides will be processed and presented by HLA molecules to elicit a potent, tumor-specific T-cell response. A narrow genomic focus on TMB is insufficient, as evidenced by malignancies like hepatocellular carcinoma where high-quality, presented neoantigens remain scarce despite detectable mutations, highlighting the discordance between mutation presence and antigen presentation (17). Integration with transcriptomic data filters candidates to those derived from genes that are actually expressed in the tumor, increasing the likelihood of targeting relevant tumor cell populations. Further refinement comes from epigenomic layers; DNA methylation patterns can silence gene expression, rendering mutations in hypermethylated promoters irrelevant for vaccine targeting. Studies have shown that pre-treatment DNA methylome and transcriptome profiles correlate with melanoma response to anti-PD1, suggesting that epigenetic regulation of immune-related pathways influences the permissive environment for therapy, which is equally critical for vaccine-primed responses (43, 79). For instance, intragenic hypermethylation of SLC22A17 is associated with better clinical outcomes in cutaneous melanoma, implicating epigenetically regulated pathways in TME modulation (80). Advanced pipelines now systematically integrate whole exome and transcriptome sequencing to define a patient-specific search space, as demonstrated in personalized vaccine design studies where raw NGS data is analyzed to select neoantigens based on a composite of antigenicity, immunogenicity, and HLA-binding affinity (81). This computational immunoinformatics approach, validated by molecular docking simulations, forms the backbone of bespoke multi-epitope vaccine construction.

Beyond neoantigen selection, multi-omics data is paramount for understanding the immune contexture of the tumor, which dictates both the feasibility of priming a *de novo* response and the potential for synergy with other immunotherapies. Bulk transcriptomic analyses have been instrumental in classifying melanoma into distinct immune subtypes with prognostic significance. Bioinformatics analyses consistently reveal immune-heterogeneous clusters, such as immune “cold” subtypes characterized by low immune cell infiltration and poor outcomes, and immune “hot” subtypes with high cytolytic activity and favorable prognosis (29, 82). These subtypes are not merely descriptive; they have direct implications for vaccine strategy. Patients with an immune-inflamed “hot” TME might benefit from vaccines designed to broaden an existing response or target subdominant clones, whereas those with “cold” tumors may require vaccines engineered to be more immunogenic or co-administered with potent immune modulators to overcome exclusion. Single-cell RNA sequencing (scRNA-seq) further deconvolutes this heterogeneity, revealing functional states of individual cell populations. For example, scRNA-seq has identified specific melanoma cell subpopulations, such as a CORO1A+ subtype, that appear more sensitive to Natural Killer (NK) and T-cell-mediated cytotoxicity, while other subtypes exhibit resistance mechanisms (83). Similarly, the analysis of cancer-associated fibroblasts (CAFs) through integrated single-cell and bulk sequencing has led to prognostic signatures that correlate with an immunosuppressive TME, characterized by reduced CD8+ T cells, which could predict poor vaccine efficacy (84). Furthermore, the identification of MITF+ T-cell and M2 macrophage sub-populations linked to prognosis through machine learning integration of multi-omics data underscores the power of these tools to discover novel cellular players influencing outcomes (85).

Patient stratification for combination therapy, particularly integrating vaccines with ICIs, demands predictive biomarkers that go beyond PD-L1 expression. Multi-omics profiling is uncovering nuanced biological states predictive of ICI response, which directly informs who might benefit most from a vaccine-ICI combination. Proteomic analysis of plasma has revealed an inverse correlation between apolipoproteins and inflammatory proteins, where a profile of low apolipoproteins and high inflammation markers pre-treatment predicts shorter progression-free survival on immunotherapy, suggesting a dysregulated, protumorigenic inflammatory state (86). At the tissue level, the activation state of specific signaling pathways serves as a potent stratifier. Research indicates that distinct patterns of IFN-γ signaling, quantified by STAT1 protein levels via multiplex immunofluorescence, are superior to PD-L1 alone in predicting response to ICIs, with STAT1high tumors showing improved outcomes (87). This is mechanistically supported by findings that mutations activating the NF-κB pathway (e.g., in NFKBIE) are enriched in responders to anti-PD1 therapy, linking genomic alteration to a pro-inflammatory tumor cell phenotype conducive to immune attack (88). For vaccines, the metabolic state of antigen-presenting cells is equally crucial. A detailed multi-omics analysis of DCs vaccines revealed that a metabolic profile skewed towards glycolysis and associated with increased MCT1 expression correlates with suppressed immunostimulatory function and worse patient overall survival (53). Thus, pre-vaccination assessment of DC metabolic competence or circulating myeloid precursors could stratify patients likely to mount a robust vaccine-induced response.

Longitudinal monitoring through liquid biopsy represents a dynamic dimension of multi-omics integration, enabling real-time assessment of treatment efficacy and early detection of resistance. ctDNA has emerged as a robust, non-invasive biomarker for monitoring melanoma under therapy. Tumor-informed ctDNA assays, tracking patient-specific variants, show that ctDNA levels correlate with metabolic tumor volume and that an early decrease in ctDNA allele fraction is strongly associated with improved progression-free and overall survival in patients on ICI (56). A meta-analysis confirms that detectable ctDNA before and during ICI therapy is a significant prognostic marker for poorer outcomes (89). Importantly, ctDNA clearance at landmark timepoints, such as 6 months into anti-PD-1 therapy, serves as an independent predictor of long-term clinical benefit and could guide personalized treatment duration decisions (90). This serial monitoring can be combined with other liquid biomarkers. A multiplatform approach integrating ctDNA analysis with circulating melanoma cell count and copy number variation profiling can track clonal evolution and identify emerging resistance mutations to targeted therapies like BRAF/MEK inhibitors (91). Beyond nucleic acids, the integration of microbiomic and metabolomic data from fecal samples adds another layer. The gut microbiota composition and associated metabolome at baseline differ between responders and non-responders to both immunotherapy and targeted therapy, with specific taxa and metabolites like anethole associated with response, while others like butyrate esters correlate with non-response and decreased survival (92, 93). This suggests that modifiable host factors could be targeted to improve therapeutic outcomes, creating a rationale for combining vaccines with microbiome-modulating interventions.

The clinical implementation of integrative frameworks for neoantigen vaccine design is primarily hindered by significant technical hurdles stemming from the inherent heterogeneity of multi-omics data (94, 95). Different high-throughput platforms, such as next-generation sequencing and mass spectrometry, produce data with fundamentally distinct measurement scales—ranging from discrete genomic counts to continuous metabolomic values—and varying patterns of missing values (96, 97). Consequently, rigorous data standardization and normalization are essential pre-processing steps to ensure cross-layer comparability and prevent technical noise from overwhelming genuine biological signals (98). Furthermore, the elimination of batch effects—systemic technical variations introduced by different experimental runs, operators, or platforms—is critical for preserving true biological variance during cross-modal association analysis (99). Beyond pre-processing, a central decision in the integration process is the strategic assignment of weights to different omics datasets. Simply treating all modalities equally in early integration (data concatenation) can lead to models being dominated by high-dimensional, noisy datasets, thereby masking subtle but vital signals from other layers (97, 98). To address these issues, current methodological solutions have shifted toward intermediate and late integration strategies. Statistical frameworks such as Multi-Omics Factor Analysis (MOFA+) utilize variational inference and sparsity constraints to reconstruct low-dimensional representations and identify key cross-modal drivers (100). Additionally, advanced machine learning tools (e.g., DIABLO) and deep learning architectures, such as autoencoders and multi-view networks, are increasingly employed to adaptively weight data sources and extract complex non-linear features (101–104). These sophisticated computational strategies enable more robust patient stratification and precise vaccine response prediction by distilling non-redundant biological signals from heterogeneous high-throughput data.

In conclusion, the integration of multi-omics data transforms personalized neoantigen vaccination from a bespoke mutation-targeting exercise into a holistic therapeutic strategy. It informs which antigens to target, predicts the capacity of the host immune system to respond, identifies optimal combination partners, and provides the tools for dynamic adaptation of therapy based on real-time molecular feedback. While challenges in data standardization, computational complexity, and clinical validation remain, this integrative approach is essential for selecting the right patient for the right vaccine at the right time, and for combining it with the right adjuvant therapy to overcome resistance and achieve durable clinical remission in melanoma.

## 5 Overcoming resistance: rationale for combining neoantigen vaccines with immunotherapy and targeted agents

The holistic, multi-omics-informed strategy for personalized neoantigen vaccine design directly confronts the core challenge in melanoma therapy: therapeutic resistance. While neoantigen vaccination aims to generate a *de novo* or amplified tumor-specific T-cell response, this response can be suppressed, exhausted, or evaded by the sophisticated defense mechanisms of the tumor and its microenvironment (3, 105). Therefore, the integration of neoantigen vaccines with other therapeutic modalities is not merely additive but fundamentally synergistic, designed to dismantle multiple layers of resistance simultaneously and convert immunologically “cold” or immunosuppressive tumors into “hot,” immunologically responsive ones (105, 106). This combinatorial rationale is driven by an evolving understanding of tumor-intrinsic, microenvironmental, and systemic barriers to effective immunity.

A primary rationale for combination lies in overcoming the profound immunosuppression mediated by the TME. Monotherapy with ICIs like anti-PD-1 and anti-CTLA-4 has revolutionized melanoma care, yet innate and acquired resistance limit their efficacy for a substantial proportion of patients (72, 105). Resistance mechanisms include upregulation of alternative immune checkpoints, recruitment of immunosuppressive cells like regulatory T cells and MDSCs, and metabolic alterations that inhibit T-cell function (3, 105). A neoantigen vaccine can address a key limitation of ICI monotherapy—the prerequisite of a pre-existing but suppressed T-cell repertoire—by actively priming and expanding tumor-specific T-cell clones (107). These newly mobilized T cells, specific for patient-unique neoantigens, then infiltrate the tumor, where their effector function can be protected and sustained by concurrent ICI blockade of inhibitory signals like PD-1. This sequence is supported by the concept that effective immunotherapy requires both a potent antigenic signal (provided by the vaccine) and the removal of T-cell inhibition (provided by ICIs) (26). Furthermore, the neoadjuvant setting provides a powerful platform for such combinations, allowing for *in vivo* assessment of biological and pathological response to the vaccine-ICI regimen and enabling biomarker discovery from serial tumor samples (107, 108).

**Figure 3:**
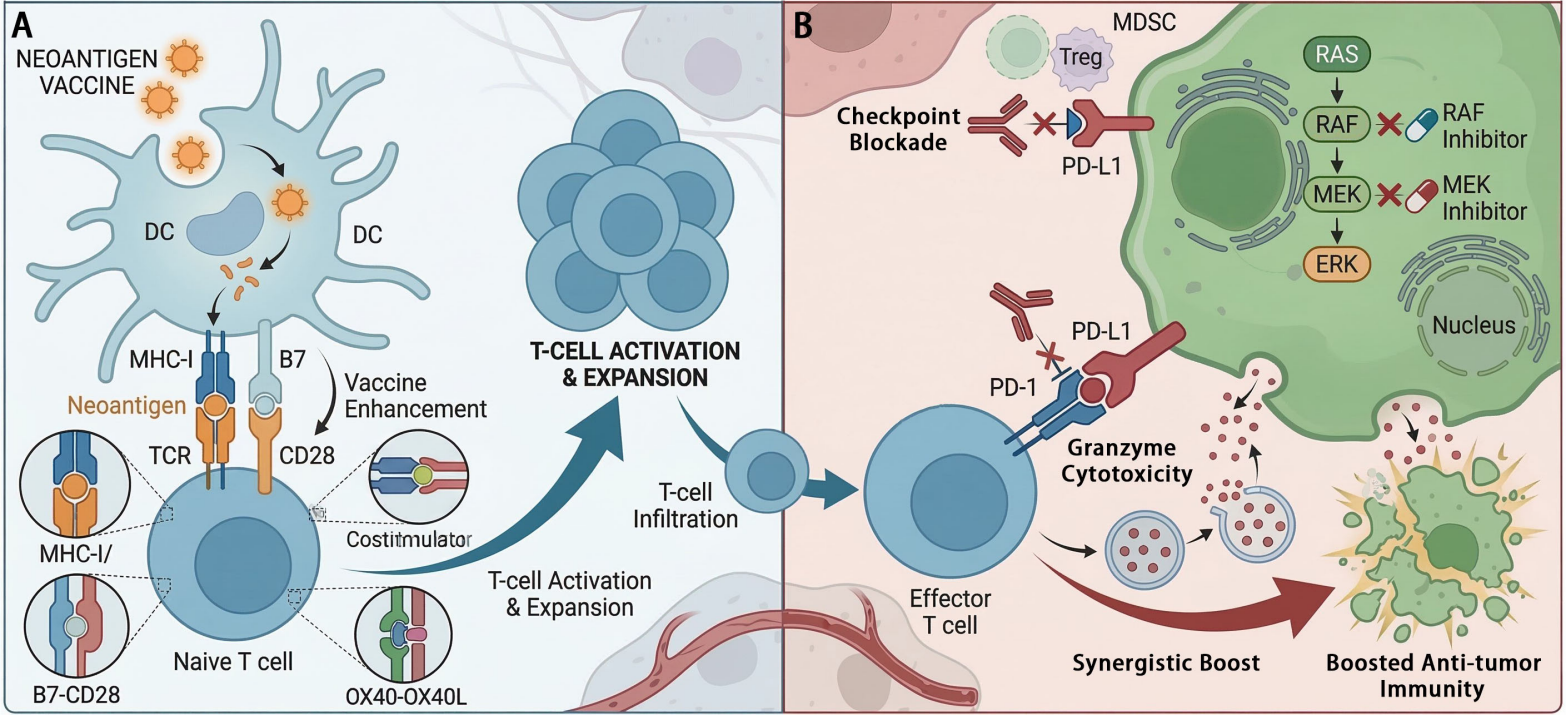
Molecular mechanisms of synergistic interaction between neoantigen vaccines and ICIs. **(A)** Vaccine-enhanced priming: Personalized neoantigen vaccines provide a potent antigenic signal, enabling DCs to prime and expand a *de novo* repertoire of tumor-specific naive CD8+ T cells via MHC-I/TCR interaction and co-stimulatory signaling (e.g., CD28/B7). **(B)** Combined inhibition: Concurrent administration of ICIs (e.g., anti-PD-1, anti-CTLA-4) removes inhibitory “brakes,” restoring the effector function of exhausted T cells and protecting newly infiltrated vaccine-induced T cells. Simultaneously, targeted agents (e.g., BRAF/MEK inhibitors) modulate the tumor cell’s intrinsic signaling, promoting immunogenic remodeling and increasing antigen presentation.

Combining neoantigen vaccines with molecularly targeted agents, particularly for BRAF-mutant melanoma, represents another strategically sound approach to modulate the TME and overcome resistance. BRAF/MEK inhibitor combinations induce rapid tumor regression, but responses are often transient due to the emergence of resistance (106). Importantly, targeted therapy can have immunomodulatory effects, such as increasing tumor antigen expression, reducing immunosuppressive cytokine secretion, and enhancing T-cell infiltration, thereby creating a window of opportunity for immunotherapy (106, 109). However, these effects are often incomplete or unsustained. A personalized neoantigen vaccine administered concurrently or sequentially with targeted therapy could capitalize on this immunogenic remodeling by directing the emerging immune response against a broad set of clonal neoantigens, potentially delaying or preventing the outgrowth of resistant clones (106). This strategy seeks to convert the typically cytoreductive effect of targeted therapy into a more durable immunologic control (**Figure 3**). Evidence suggests that the response to targeted therapy itself may be influenced by host factors like the gut microbiota and specific immune gene expression patterns, highlighting the complex interplay between targeted agents and the immune system that combination therapies aim to harness (92).

Beyond ICIs and MAPK pathway inhibitors, the combinatorial landscape extends to novel agents that target complementary resistance pathways. Epigenetic modifiers are emerging as potent partners. For instance, inhibition of the histone methyltransferase G9a was shown to upregulate the immunogenic protein LC3B, augment the efficacy of checkpoint blockade, and induce melanoma cell death, suggesting that combining such epigenetic drugs with neoantigen vaccination could enhance antigen presentation and tumor immunogenicity (110). Similarly, CDK4/6 inhibitors, known to induce tumor cell senescence and modulate the TME, are being investigated in combination with both immunotherapy and targeted therapy in melanoma, with preclinical data supporting their role in enhancing antitumor immune responses (111). Another promising avenue involves targeting DNA repair pathways. Research demonstrates that MAPK inhibitor-resistant melanoma cells can exhibit low expression of the DNA damage sensor ATM, conferring sensitivity to PARP inhibitors (112). This creates a synthetic lethal interaction when PARP inhibitors are combined with MAPK inhibitors, an approach that could be integrated with vaccination to target residual disease and resistant subclones (112). These examples illustrate the principle of using non-immunotherapies to create a TME more permissive to vaccine-induced T-cell activity.

The rationale also encompasses innovative immunotherapeutic formats. Bispecific T-cell engagers, like tebentafusp for uveal melanoma, demonstrate that redirecting T cells to TAAs (in this case, gp100) is efficacious (113). A neoantigen vaccine could potentially be combined with such agents to amplify the pool of T cells capable of being redirected, or to target antigens beyond the limited HLA-restricted ones targeted by bispecifics. Furthermore, modalities like photodynamic therapy (PDT) have been shown to induce immunogenic cell death and, when combined with a model tumor vaccine and anti-PD-1, potentiate systemic antitumor immunity in preclinical models, outlining a paradigm where localized tumor destruction enhances systemic vaccination effects (26).

Translating these rationales into clinical practice requires careful consideration of sequencing, toxicity, and biomarker-guided patient selection. The multidisciplinary team (MDT) approach is critical for navigating these complexities, integrating insights from genomic profiling, immune monitoring, and clinical parameters to tailor combination regimens (109). Biomarkers will be indispensable for identifying which patients are most likely to benefit from specific combinations. For example, transcriptional signatures associated with proliferation (E2F targets, G2M checkpoint) versus immune activation (allograft rejection, IFN-γ response) have been differentially correlated with outcomes to targeted therapy and immunotherapy, respectively, and could inform whether to prioritize a vaccine-plus-targeted or vaccine-plus-immunotherapy approach (114). Similarly, on-treatment changes in gut microbiota or circulating immune markers may provide early signals of response or emerging resistance, allowing for dynamic adaptation of the therapeutic strategy (72, 92). As the field progresses, the ultimate goal is to move beyond empirical combinations to rational, biomarker-driven integration where neoantigen vaccines serve as the foundational immunologic primer within a multi-modal framework designed to preempt and overcome the heterogeneous mechanisms of resistance in melanoma (105, 115) (**Table 3**).

**Table 3:** Mechanistic rationales for rational combination strategies in melanoma.

Combination strategy	Synergistic mechanism (biological rationale)	Primary resistance addressed
Vaccine + ICIs (e.g., Anti-PD-1)	Vaccine expands tumor-specific T-cell pool; ICIs remove inhibitory signals to unleash effector function	Prerequisite of a pre-existing but suppressed T-cell repertoire; T-cell exhaustion
Vaccine + Targeted Therapy (BRAFi/MEKi)	Targeted agents induce immunogenic remodeling, increase antigen expression, and enhance T-cell infiltration	Transient response to targeted therapy; outgrowth of resistant clones; immunosuppressive cytokine secretion
Vaccine + Epigenetic Modifiers (e.g., G9a inhibitor)	Inhibition of G9a upregulates immunogenic proteins (e.g., LC3B) and enhances antigen presentation	Epigenetic silencing of immune-related pathways and tumor immunogenicity
Vaccine + DNA Repair Inhibitors (PARPi)	MAPK inhibitor-resistant cells exhibit low ATM expression, creating a synthetic lethal interaction with PARPi	Survival of residual disease and resistant subclones following MAPK pathway inhibition
Vaccine + Microbiome Modulation	Targeting host factors (gut microbiota) to modulate systemic immunity and ICI efficacy	Host-related systemic immunosuppression and differential clinical response based on metabolic fitness

## 6 Technological frontiers: AI, novel platforms, and emerging delivery systems

The transition towards rational, biomarker-driven combination therapies is fundamentally enabled by concurrent technological revolutions that are reshaping every facet of personalized immuno-oncology, from neoantigen discovery and vaccine formulation to treatment monitoring and response assessment. At the nexus of these advancements lie AI and machine learning, which are becoming indispensable for deciphering the immense complexity of multi-omics data to inform clinical decisions (116, 117). AI algorithms are increasingly applied to integrate genomic, transcriptomic, proteomic, and radiomic datasets, identifying patterns beyond human discernment to refine neoantigen prediction, optimize vaccine design, and discover novel predictive biomarkers (81). For instance, machine learning frameworks have been employed to construct immune cell-related prognostic signatures in skin cutaneous melanoma, with derived models demonstrating superior performance in predicting both overall survival and response to anti-PD-1 immunotherapy compared to numerous existing signatures (117). Beyond genomics, AI is profoundly impacting clinical imaging and histopathology. In radiomics, machine learning models analyzing pretreatment CT textures have shown high accuracy in predicting overall survival and treatment response in metastatic melanoma patients receiving immunotherapy (118). Furthermore, dual-energy CT (DECT)-derived quantitative metrics, such as tumor iodine concentration, offer a functional imaging biomarker; early changes in iodine concentration have been validated to improve the identification of delayed responders and differentiate true progression from pseudoprogression in patients treated with ICIs (119). Automated, AI-driven tools for detecting and segmenting metabolically active melanoma lesions on whole-body ¹^8^F-FDG PET/CT scans are also emerging, facilitating the precise quantification of metabolic tumor burden, a promising biomarker for immunotherapy outcomes (120). Remarkably, pre-treatment metabolic patterns in specific brain networks, such as the cognition/language network assessed via ¹^8^F-FDG PET, have been associated with treatment response and survival in oncology, including melanoma patients receiving anti-PD-1 therapy, suggesting a complex, systemic interplay between neuro-metabolic states and anti-tumor immunity (121). In digital pathology, deep learning algorithms enable the automated, reproducible, and spatially resolved quantification of TILs across whole-slide images, moving beyond subjective manual assessment to provide a comprehensive map of the tumor immune microenvironment as a powerful predictive tool for immunotherapy (122). Complementing these digital pathology advancements, spatial transcriptomics (ST) has emerged as a transformative frontier, enabling high-throughput whole-transcriptome profiling while preserving the precise spatial architecture of the melanoma TME (123). ST allows for the high-resolution mapping of the ‘spatial landscape of immunoediting,’ revealing how tumor-immune interactions—such as the formation of inhibitory environments along the tumor-stromal interface—evolve dynamically across millimeter scales (124). In metastatic settings, state-of-the-art ST applications have identified unique organ-specific niches, such as the neuron-like cell states and chromosomal instability in brain metastases (125), and the SERPINA3-mediated pro-tumorigenic stroma in leptomeningeal disease (126). Furthermore, the integration of ST with single-cell RNA sequencing (scRNA-seq) has become a standard strategy to resolve complex ‘spatial niches’ and cell-type functional states (125, 127). Beyond transcriptomics, the convergence of spatial multi-omics, such as Digital Spatial Profiling (DSP), has facilitated the discovery of predictive biomarkers, including the finding that PD-L1 expression on macrophages (CD68+ cells), rather than tumor cells, is a superior indicator of immunotherapy response (128). This spatial contexture is vital for optimizing personalized neoantigen vaccines, as it identifies the specific niches that primed T cells must navigate and infiltrate to exert anti-tumor effects (123, 125, 126, 129).

Parallel to the analytical power of AI, revolutionary advances in vaccine platforms and delivery systems are addressing historical limitations of immunogenicity and precise targeting. EVs, particularly exosomes, have surged as a highly versatile frontier. These natural nanoscale carriers offer inherent biocompatibility, low immunogenicity, and the ability to cross biological barriers, making them ideal for drug and antigen delivery (59, 130). Engineered exosomes can be loaded with various cargoes, including peptide neoantigens, siRNAs, miRNAs, and chemotherapeutic agents (59, 131). A pivotal innovation is the concept of “neoantigen-painted” exosomes, where peptide neoantigens are anchored onto the surface of serum-derived exosomes. This approach promotes lymph node homing and enhances DCs uptake, leading to significantly amplified antigen-specific CD8+ T cell responses *in vivo* and, when combined with anti-PD-1 therapy, achieving complete tumor remission in murine models (23). Beyond their therapeutic utility, tumor-derived EVs are a rich source of liquid biopsy biomarkers. Melanoma-derived exosomes (MEXs) carry tumor-specific proteins and genomic material, reflecting the real-time state of the tumor and its microenvironment (130, 132). Circulating small EVs expressing PD-L1 (sEV-PD-L1) are being investigated as dynamic biomarkers; a decrease in sEV-PD-L1 levels has been observed during pseudoprogression in a melanoma patient on immunotherapy, offering a potential tool to distinguish this phenomenon from true progression and prevent premature therapy cessation (61, 133). The EV-mediated crosstalk between melanoma cells and the immune system is a critical modulator of tumor progression and therapy response, underscoring their dual role as therapeutic vectors and biomarkers (134).

Nanotechnology provides another expansive platform for optimizing vaccine design and delivery. Studies systematically investigating parameters for cancer nanovaccines have identified optimal nanoparticle sizes (≤400 nm) and adjuvant combinations that can cure most tumor-bearing mice across several cancer types, including melanoma (135). Single-cell sequencing of immune cells from vaccinated mice has further revealed distinct receptor diversity and biomarker profiles (e.g., KLRG1, S100A4) associated with response, informing future vaccine optimization (135). Novel protein nanoparticle platforms are also emerging. For example, self-assembled protein nanoparticles based on Brucella BP26 protein, genetically engineered to display tandem repeats of a melanoma neoantigen, have demonstrated the ability to elicit potent neoantigen-specific CD4+ T cell responses and inhibit tumor growth in aggressive melanoma models (22). For localized and sustained delivery, microneedle-based systems represent a minimally invasive strategy. A recent development involves hyaluronic acid-based microneedles co-encapsulating a nanomotor cancer vaccine and the chemotherapeutic doxorubicin. Upon skin application, the released nanomotor vaccine utilizes L-arginine to achieve chemotactic targeting to tumor tissues, inducing immunogenic cell death and activating antitumor immunity synergistically with chemotherapy (25). Other innovative platforms include flexible liposomal polymer complexes (LPPC) that can non-covalently adsorb various immunomodulatory proteins (e.g., anti-CD3, HLA/peptide complexes, DCs membrane proteins), creating a regulable system capable of delivering pan-T cell or antigen-specific Th1/Th2 responses as needed, thereby offering a customizable platform for personalized immunotherapy (24).

**Figure 4:**
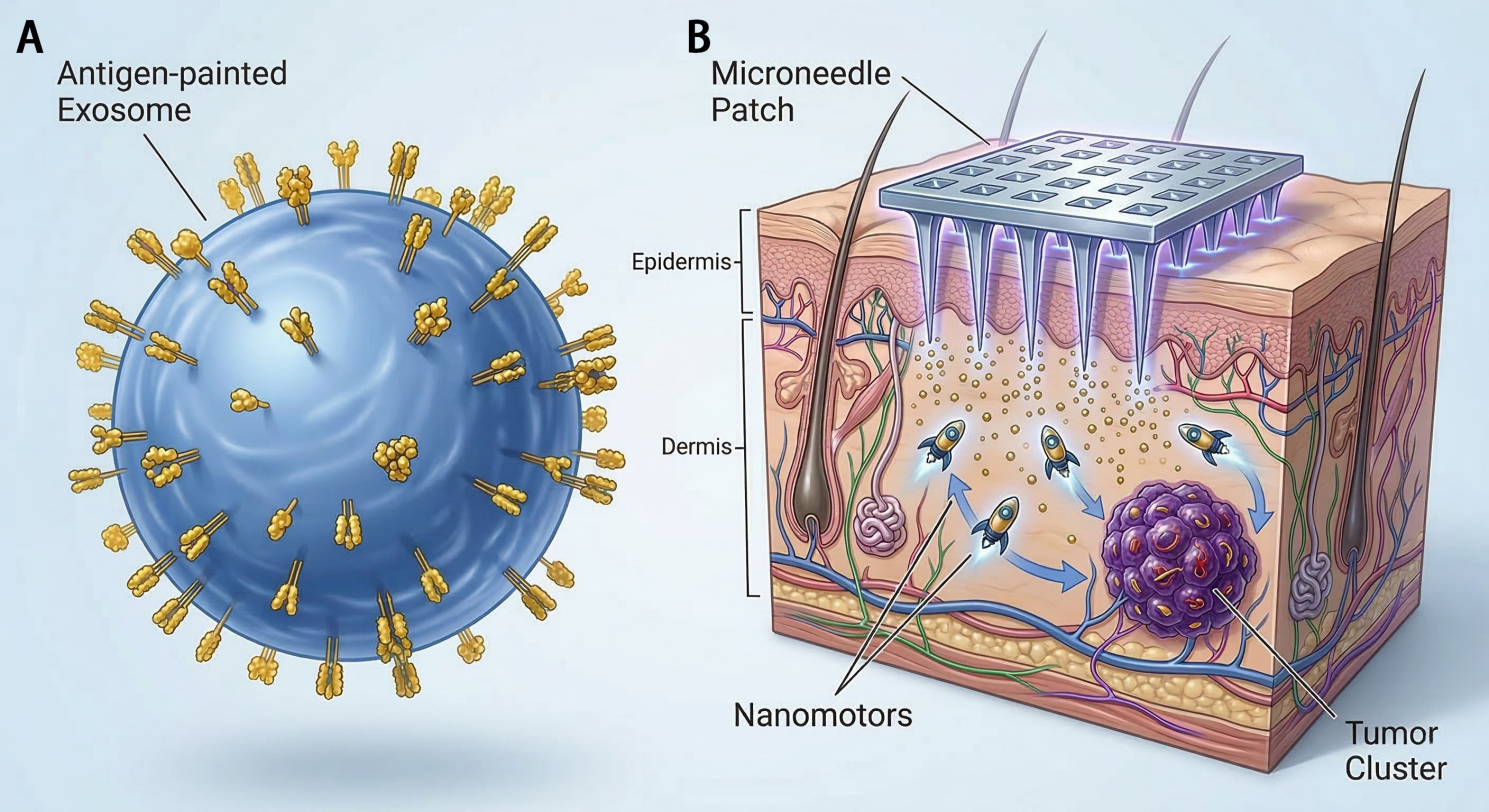
Technological frontiers in neoantigen vaccine delivery: bio-engineered exosomes and nanomotor-integrated microneedles**. (A)** Antigen-painted Exosomes: Schematic of serum-derived exosomes with surface-anchored peptide neoantigens. This bio-mimetic platform leverages innate biocompatibility and superior membrane-penetrating capabilities to promote efficient lymph node homing and DC uptake, significantly amplifying antigen-specific CD8+ T-cell responses. **(B)** Microneedle Patch and Nanomotors: Illustration of a minimally invasive transdermal delivery system. Upon application, the released nanomotor-based vaccines utilize L-arginine for chemotactic targeting toward the Tumor Cluster, inducing immunogenic cell death and activating systemic anti-tumor immunity in synergy with chemotherapy.

The therapeutic arsenal is further expanded by modalities such as antibody-drug conjugates (ADCs) and oncolytic viruses, which can be integrated into combination regimens with vaccines. An ADC targeting HER-3 (EV20/MMAF) has shown potent, specific cytotoxicity against melanoma cells and superior efficacy in preventing metastasis compared to a BRAF inhibitor in preclinical models, highlighting a targeted strategy for tumors expressing specific antigens (136). Oncolytic viruses like VSV-GP selectively infect and kill melanoma cells while stimulating antitumor immunity. Although efficacy in syngeneic models can be limited by host type I IFN responses, VSV-GP has demonstrated significant survival prolongation and reduction of lung metastases, positioning it as a candidate for viro-immunotherapy combinations (137). The convergence of these technological frontiers—AI-driven insights, smart biomaterial-based delivery systems, and novel therapeutic agents—creates a powerful toolkit. AI not only guides the initial design of personalized vaccines from NGS data (81) but also interprets complex biomarker signals from advanced imaging (119–121), liquid biopsies (61, 133), and histopathology (122) to monitor therapy and adapt strategies. Meanwhile, next-generation platforms like engineered exosomes (23) and nanovaccines (22, 135) ensure that the designed therapeutic constructs are delivered with high efficiency and immunogenicity. Together, these innovations are forging a path toward truly adaptive and precise immuno-oncology, where treatment is continuously informed by a dynamic stream of multi-modal data and implemented through sophisticated, targeted delivery systems (**Figure 4**).

## 7 Clinical translation: trial design, regulatory pathways, and implementation challenges

The convergence of sophisticated vaccine platforms, multi-omics biomarker discovery, and AI creates a powerful toolkit for precision immuno-oncology. However, translating these scientific and technological advances into routine clinical practice necessitates navigating complex pathways of trial design, regulatory approval, and healthcare system implementation. The integration of personalized neoantigen vaccines into the established therapeutic landscape for melanoma, particularly in the context of multimodal biomarker monitoring, presents unique opportunities and formidable challenges that must be systematically addressed.

The design of clinical trials evaluating neoantigen vaccines has evolved from early-phase safety studies to more nuanced investigations that reflect their role as immunomodulators rather than standalone cytoreductive agents. Given the established efficacy of ICIs as standard-of-care in both metastatic and adjuvant settings (4, 8, 138, 139), contemporary trials predominantly investigate vaccine-ICI combinations. A critical design consideration is the sequencing and timing of vaccine administration. The neoadjuvant setting, where therapy is delivered prior to surgical resection, has emerged as a particularly informative paradigm (107, 108). This model allows for the assessment of pathological response as a primary endpoint and provides unparalleled access to longitudinal biospecimens for biomarker discovery, enabling researchers to mechanistically understand treatment responsiveness and resistance (107, 108, 140). Trial designs are increasingly incorporating novel endpoints that reflect the biological action of vaccines. For instance, the phase II KEYNOTE-D36 trial for the personalized neoepitope vaccine EVX-01 combined with pembrolizumab employs a novel endpoint: evaluating if the vaccine can improve the best overall response in patients who initially achieve only stable disease or a partial response to pembrolizumab monotherapy (21). This “rescue” or “deepening of response” endpoint is strategically chosen to provide a decisive clinical readout on the vaccine’s additive value before committing to larger phase III investments (21). Furthermore, the exploration of vaccines in rare melanoma subtypes with unmet needs, such as uveal or mucosal melanoma, requires tailored trial designs that account for distinct biology and lower TMB, as evidenced by investigations into neoadjuvant ICI for conjunctival melanoma (141) and adoptive cell therapy for uveal melanoma (142).

A cornerstone of clinical translation is the development and validation of predictive and monitoring biomarkers to guide patient selection and treatment decisions. While traditional biomarkers like PD-L1 immunohistochemistry have shown inconsistent predictive value in melanoma (8, 143), the integration of multi-omics approaches is yielding more robust tools. ctDNA has solidified its role as a critical liquid biopsy biomarker. In the adjuvant setting, baseline ctDNA detection identifies patients with resected stage III melanoma at the highest risk of relapse and predicts worse overall survival (57, 144). Longitudinally, ctDNA dynamics offer real-time, non-invasive monitoring of treatment efficacy; clearance of ctDNA correlates with clinical response and prolonged survival during ICI therapy (89, 90, 145), while persistent detection often heralds disease progression (57, 90). This utility extends to the challenging scenario of pseudoprogression, where conventional imaging can be misleading. Monitoring biomarkers like PD-L1 expression on circulating small extracellular vesicles (sEV-PD-L1) may help distinguish true progression from inflammatory pseudoprogression, preventing the premature discontinuation of effective therapy (133, 146). Beyond ctDNA, other systemic and tumoral immune profiles are under investigation. Pretreatment levels of chemokines like CXCL9 and CTACK (140), frequencies of S100A9+ monocytes in peripheral blood (147), and quantitative assessment of TILs, particularly CD8+ T cell density, have been associated with ICI response (148). Even host physiological factors, summarized as allostatic load, are being linked to both immune-related toxicity and survival outcomes (149). The challenge lies in moving from correlative observations to validated, standardized assays that can function as companion diagnostics within regulatory frameworks.

The implementation of personalized vaccine therapies into mainstream oncology practice faces significant logistical and economic hurdles. The very premise of personalization—designing a unique therapeutic construct for each patient based on their tumor’s mutanome—introduces complexities in manufacturing, quality control, and turnaround time. This process, from tumor sequencing and neoantigen prediction to vaccine synthesis and release testing, must be streamlined to fit within a clinically viable window, especially in the neoadjuvant setting where treatment duration is limited before surgery (108). Furthermore, effective application requires a robust MDT approach. Optimal patient care hinges on collaboration between surgical, medical, and dermatologic oncologists, pathologists, radiologists, and translational scientists (109). The MDT is essential for accurate staging, timing of biomarker testing (e.g., BRAF mutation status), interpretation of complex response patterns on imaging (4), and management of unique toxicities from combination regimens (8, 109). Radiologists, in particular, must be familiar with novel response patterns and the role of imaging biomarkers, such as subcutaneous adipose tissue metrics which have been linked to ICI response (150), and texture analysis features like tumor kurtosis that may predict overall survival (151). Real-world evidence studies, such as those characterizing melanoma management in specific populations (152) or evaluating long-term ICI monotherapy outcomes (153), highlight the gap between clinical trial efficacy and effectiveness in broader, more heterogeneous patient populations, underscoring the need for pragmatic implementation strategies.

Regulatory pathways for personalized cancer vaccines are still maturing. Unlike conventional drugs with a fixed chemical structure, autologous vaccines are biologic products derived from a patient’s own tissue, blurring the lines between drug and procedure. Regulatory agencies must establish frameworks for evaluating the safety, purity, potency, and consistency of these bespoke products. The recent approval of the first TILs therapy, lifileucel, for advanced cutaneous melanoma provides a precedent for regulating complex, personalized cell-based immunotherapies (142). This approval pathway will inform the development of regulatory standards for neoantigen vaccine platforms. Key regulatory considerations will include defining the critical quality attributes of the vaccine product, validating the bioinformatics pipelines used for neoantigen selection, and determining appropriate clinical endpoints. Society guidelines, such as those from the Society for Immunotherapy of Cancer (SITC), will play a crucial role in establishing best practices for therapy selection, toxicity management, and response assessment, thereby shaping the clinical environment into which these therapies are introduced (8).

Finally, addressing clinical challenges such as primary and acquired resistance, and optimizing combination strategies, is integral to successful translation. The rationale for combining vaccines with ICIs is strong, aiming to amplify tumor-specific T cell populations that can then be more effectively unleashed by checkpoint blockade (21). However, resistance mechanisms are multifactorial. Biomarker studies point to the immunosuppressive TME, including factors like high baseline levels of interleukin-6 (IL-6) or MDSCs (154), as well as systemic factors like elevated prostaglandin E2 (PGE2) (155). These insights directly inform the design of next-generation combination trials, pairing vaccines not only with ICIs but also with targeted agents against specific resistance pathways, such as COX-2 inhibitors to lower PGE2 (155). Furthermore, integrating vaccines with other localized modalities like oncolytic viruses or electrochemotherapy for locoregionally advanced disease could enhance antigen release and immune activation in a synergistic manner (156). The ultimate goal is to leverage multi-omics biomarkers in an iterative feedback loop: pretreatment biomarkers guide initial therapy selection, on-treatment biomarker dynamics (e.g., ctDNA, sEV-PD-L1) inform early response assessment and the need for treatment adaptation, and post-treatment biomarkers define the need for further adjuvant therapy or surveillance (57, 89, 90, 133). Successfully navigating the path from bench to bedside will require sustained collaboration across academia, industry, regulatory bodies, and healthcare providers to overcome the inherent challenges of personalization and deliver on the promise of precision immuno-oncology for all patients with melanoma.

## 8 Conclusion and future perspectives

The journey in melanoma therapy has evolved from a landscape of limited options with poor prognoses to an era defined by transformative treatments that have fundamentally altered patient trajectories. The unprecedented survival benefits conferred by ICIs and targeted BRAF/MEK inhibitors have established a new baseline from which future innovations must ascend. However, the persistent challenges of primary and acquired resistance, along with the inability to achieve durable responses in a significant proportion of patients, underscore that current therapeutic paradigms, while revolutionary, are not yet curative for all. The path forward lies in the deeper integration of precision medicine principles, moving beyond broad population-based strategies to truly individualized care. This vision centers on the interdependent symbiosis of two powerful, data-driven modalities: personalized neoantigen-directed vaccines and comprehensive multi-omics biomarker profiling. Together, they represent the next evolutionary step in immuno-oncology, aiming to amplify, direct, and sustain the anti-tumor immune response with exquisite specificity.

The rationale for personalized neoantigen vaccines is compelling, capitalizing on the genetic heterogeneity of melanoma to target truly tumor-specific mutations. The transition from shared TAAs to patient-specific neoantigens marks a critical advance in vaccine design, potentially reducing off-target effects and enhancing immunogenicity. Platforms utilizing SLPs and, more recently, mRNA have demonstrated the ability to induce robust, antigen-specific T-cell responses. The true potential of these vaccines is likely unlocked not as monotherapies but as integral components of combination regimens designed to overcome the immunosuppressive TME. Their role is particularly promising in earlier-stage or minimal residual disease settings, such as the adjuvant and neoadjuvant spaces, where an intact immune system and lymphatic architecture can be optimally primed. Here, vaccines can act as “accelerators” to expand tumor-specific T-cell clones, which can then be more effectively unleashed by concomitant or sequential ICI therapy, creating a powerful immune synergy. This approach aims to convert immunologically “cold” tumors into “hot” ones, broadening the population of patients who benefit from immunotherapy.

The successful implementation of this strategy is inextricably linked to the parallel advancement of biomarker science. Precision immuno-oncology demands a shift from reactive to predictive and adaptive treatment models, guided by dynamic biological data. Multi-omics profiling—integrating genomic, transcriptomic, proteomic, and immunophenotypic data—provides the granularity needed to decipher the complex interplay between tumor genetics and host immunity. This integrated analysis can inform every stage of the therapeutic journey: from pretreatment selection of optimal neoantigens and prediction of ICI responsiveness, to real-time monitoring of treatment efficacy and early detection of resistance through tools like ctDNA. The future of patient management will rely on these iterative biomarker feedback loops. For instance, a lack of vaccine-induced T-cell expansion or a rising ctDNA level during therapy could trigger an adaptive intervention, such as switching or adding a combinatorial agent targeting a specific resistance pathway identified through ongoing profiling. This dynamic approach moves away from fixed-duration therapy towards biologically informed treatment adaptation, a cornerstone of next-generation personalized care.

Looking ahead, the quest for a curative synergy will focus on several key frontiers. First, optimizing combination regimens is paramount. This extends beyond vaccine-ICI pairs to rational triplets incorporating targeted therapies (e.g., BRAF/MEK inhibitors for BRAF-mutant disease), agents modulating specific immunosuppressive pathways (e.g., COX-2 inhibitors), or localized modalities like oncolytic viruses to enhance antigen release and inflammatory signals. Second, technological innovation must continue to reduce the cost and turnaround time for neoantigen identification and vaccine manufacturing to make this paradigm accessible. AI and machine learning will play an increasingly vital role in refining neoantigen prediction algorithms, interpreting complex multi-omics datasets, and identifying novel predictive biomarker signatures. Third, novel therapeutic platforms, such as bispecific T-cell engagers (exemplified by tebentafusp for uveal melanoma) and next-generation adoptive cell therapies, will expand the arsenal of tumor-specific immunotherapies, potentially used in sequence or combination with vaccine-primed immune responses.

Clinical translation and implementation pose significant but surmountable challenges. The design of clinical trials must evolve to accommodate highly personalized therapies and incorporate biomarker-driven adaptive protocols. Regulatory frameworks need to adapt to the unique characteristics of “bespoke” medicinal products. Ultimately, the effective delivery of this complex, data-intensive care model will require a robust (MDT) approach, integrating surgical, medical, and radiation oncologists with dermatologists, pathologists, bioinformaticians, and specialized nursing teams. The MDT is essential for ensuring accurate biomarker testing at critical decision points, coordinating complex treatment sequences, and managing toxicities to preserve patient quality of life.

In conclusion, the management of melanoma stands at the threshold of a new era defined by curative ambition. The synergistic integration of personalized neoantigen vaccines, designed to educate and expand the immune repertoire, with comprehensive multi-omics biomarker profiling, which guides intelligent treatment selection and adaptation, forms the core of this future vision. This strategy seeks to address the fundamental challenges of heterogeneity and immune evasion that limit current therapies. While hurdles in manufacturing, cost, and clinical integration remain, the accelerating pace of technological and biological discovery is rapidly providing solutions. The goal is no longer merely to control advanced disease but to prevent recurrence in high-risk settings and, ultimately, to achieve durable, treatment-free remissions that equate to cure for a growing majority of patients. By relentlessly pursuing this synergy between precise immune intervention and deep biological insight, the melanoma community is poised to translate the remarkable progress of the last decade into even more transformative outcomes in the years to come.
